# Observation of extremely efficient terahertz generation from mid-infrared two-color laser filaments

**DOI:** 10.1038/s41467-019-14206-x

**Published:** 2020-01-15

**Authors:** Anastasios D. Koulouklidis, Claudia Gollner, Valentina Shumakova, Vladimir Yu. Fedorov, Audrius Pugžlys, Andrius Baltuška, Stelios Tzortzakis

**Affiliations:** 1 Institute of Electronic Structure and Laser (IESL), Foundation for Research and Technology - Hellas (FORTH), P.O. Box 1527, GR-71110 Heraklion, Greece; 2 Photonics Institute, TU Wien, Gusshausstrasse 27-387, A-1040 Vienna, Austria; 3 Science Program, Texas A&M University at Qatar, P.O. Box 23874, Doha, Qatar; 4 P.N. Lebedev Physical Institute of the Russian Academy of Sciences, 53 Leninskiy Prospekt, 119991 Moscow, Russia; 5 Center for Physical Sciences & Technology, Savanoriu Ave. 231, LT-02300 Vilnius, Lithuania; 6 Department of Materials Science and Technology, University of Crete, GR-71003 Heraklion, Greece

**Keywords:** Ultrafast lasers, Terahertz optics

## Abstract

Extreme nonlinear interactions of THz electromagnetic fields with matter are the next frontier in nonlinear optics. However, reaching this frontier in free space is limited by the existing lack of appropriate powerful THz sources. Here, we experimentally demonstrate that two-color filamentation of femtosecond mid-infrared laser pulses at 3.9 μm allows one to generate ultrashort sub-cycle THz pulses with sub-milijoule energy and THz conversion efficiency of 2.36%, resulting in THz field amplitudes above 100 MV cm^−1^. Our numerical simulations predict that the observed THz yield can be significantly upscaled by further optimizing the experimental setup. Finally, in order to demonstrate the strength of our THz source, we show that the generated THz pulses are powerful enough to induce nonlinear cross-phase modulation in electro-optic crystals. Our work paves the way toward free space extreme nonlinear THz optics using affordable table-top laser systems.

## Abstract (web summary)

Powerful terahertz pulses are generated during the nonlinear propagation of ultrashort laser pulses in gases. Here, the authors demonstrate efficient sub-cycle THz pulse generation by using two-color midinfrared femtosecond laser filaments in ambient air.

## Introduction

Terahertz (THz) radiation belongs to one of the most interesting and less explored regions of the electromagnetic spectrum which is located in-between microwave and infrared frequencies. Due to a number of unique properties, THz waves attract a lot of attention. For example, many materials like plastics, wood, paper, and clothing are transparent to THz radiation. What is more exciting, THz photons have very low energy and, unlike x-rays, do not damage tested materials. As a result, THz radiation can be successfully applied for medical diagnostics, industrial quality control, food inspection, homeland security, and others^1–4^. Moreover, THz waves are of great interest for purely scientific applications, since they can directly probe vibrational and rotational transitions, dynamics of free carriers and phonon resonances^5–7^.

Despite the rapid development of THz science during the last two decades, the majority of available THz sources remains rather weak. With the existing THz intensities the interactions of THz radiation with matter are mostly limited in the realm of linear optics, while nonlinear free space THz optics stays largely out of reach. Presently, for reaching higher field amplitudes, local field enhancement techniques are used^8,9^. On the other hand, creation and development of powerful THz sources will open the way to many exciting applications spanning from switching and controlling of magnetic domains^10–12^ to THz-enhanced attosecond pulse generation^13,14^ and table-top electron acceleration^15,16^.

Currently, the most powerful table-top THz sources are based on either optical rectification in electro-optic crystals^17–19^ or two-color filamentation in gases and liquids^20–24^. With optical rectification THz pulses with energy up to 0.9 mJ^18^ and THz conversion efficiency (ratio of generated THz energy to the input laser pulse energy) up to 3.7%^25^ were generated. Unfortunately, the optical damage threshold of electro-optic crystals prevents a significant increase of these values. Also, THz pulses generated by optical rectification are long (several picoseconds) and their spectra are narrow (below 5 THz). In turn, two-color filamentation of near infrared laser pulses offers less energetic THz pulses (up to 30 μJ in gases^22^ and up to 80 μJ in liquids^24^) with a lower THz conversion efficiency (~0.01%). Nevertheless, since the gas or liquid media recover in-between laser shots, there is no issue with the optical damage threshold. Moreover, THz pulses generated by two-color filamentation can be ultrashort (tens of femtoseconds) with a corresponding spectral bandwidth exceeding 50 THz^26,27^. Therefore, despite of the lower energy, such THz pulses allow one to achieve very high peak powers, which are necessary for studies of nonlinear interactions. Furthermore, with two-color filamentation it is possible to generate THz radiation remotely, solving propagation problems like diffraction and high absorption in atmospheric water vapor^28–31^. Note that here we are focusing on THz sources with central frequency within the well-accepted THz range of 0.1–10 THz, while above 10 THz central frequency sources with high powers have been shown in the past^32^.

At the present time, because of their abundance, Ti:sapphire lasers with central wavelength of 0.8 μm dominate as drivers of THz generation through two-color filamentation. Nevertheless, experiments with laser pulses at longer wavelengths have shown an increase of THz conversion efficiency, almost by one order of magnitude (up to 0.1% for 1.8 μm laser pulses)^33^. Although the first experiments^33^ were showing a positive trend in the near infrared, at longer wavelengths the THz efficiency was dropping again. A similar one order of magnitude improvement of the THz yield was theoretically predicted for laser pulses with 2 μm wavelength^34,35^. Particle in Cell (PIC) simulations (without propagation effects) of single color laser pulses focused into a gas jet also demonstrated growth of the THz emission when the laser wavelength was increased from 1 to 4 μm^36^. By taking into account that nonlinear propagation plays a major role in filament-based THz sources, recently we performed theoretical investigations and numerical simulations which predict that two-color filamentation of mid-infrared laser pulses can be a source of single cycle THz pulses with multi-millijoule energies and extremely high THz conversion efficiencies, which are more than two orders of magnitude higher than for 0.8 μm laser pulses^37,38^. These results were later confirmed numerically by Nguyen et al.^39^ Additionally, we showed that there is an optimal wavelength for two-color filamentation-induced THz sources: for 3.2 μm laser pulses the THz conversion efficiency reaches its maximum of about 7%^40^. Our theoretical analysis reveled that the extreme efficiency of mid-infrared two-color filamentation for THz generation is caused by a combination of many factors: strong photocurrents due to larger ponderomotive forces, longer and wider plasma channels, negligible walk-off between the fundamental and second harmonic, additional field symmetry breaking by generated high harmonics^37,38^.

Here, we provide the experimental evidence that such intense THz pulses can be generated. For the experimental verification of the enhancement of THz generation in the mid-infrared spectral range we use the recently developed powerful laser source operating at the central wavelength of 3.9 μm^41–43^. In our experiments with mid-infrared two-color laser filaments we observe the generation of submillijoule single cycle THz pulses with unprecedented THz conversion efficiency of 2.36% that exceeds by far any previously reported experimental values for plasma-based THz sources. Moreover, our numerical simulations show that by further optimizations of the experimental setup one can reach even higher THz energies and THz conversion efficiencies, close to the ones we previously predicted theoretically^37,38^. Finally, in order to demonstrate the strength of our THz source, we experimentally show that the intensity of the generated THz pulses is high enough to alter the spectrum of a probe pulse through nonlinear cross-phase modulation in an electro-optic crystal.

## Results

### Experimental implementation

**Fig. 1:**
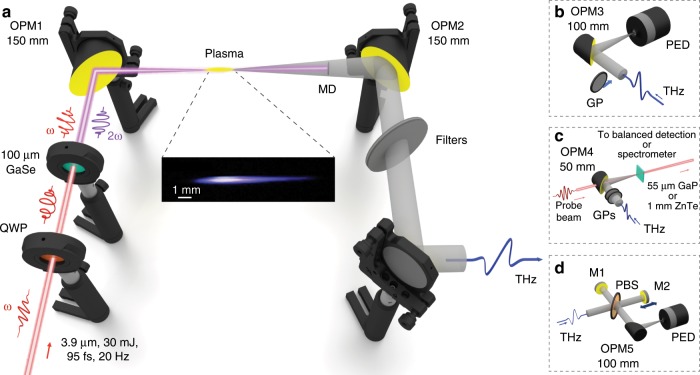
Experimental setup. **a** The setup for THz generation by two-color mid-infrared filaments. After the quarter wave plate (QWP) the 3.9 μm laser pulse passes through the gallium selenide (GaSe) crystal and generates the second harmonic pulse. The two-color laser pulse is focused by an off-axis parabolic mirror (OPM1) in ambient air and forms a filament where the THz radiation is generated. A parabolic mirror (OPM2) gathers the emitted THz pulse and guides it into one of the detection setups. The metallic disk (MD) blocks the on-axis mid-infrared radiation and generated supercontinuum, while the conically emitted THz radiation passes around it. A set of longpass filters filter out the remained unwanted radiation and also prevent the saturation of the pyroelectric detector (PED) by the intense THz pulses. **b**–**d** THz detection setups: **b** The parabolic mirror (OPM3) focuses the THz pulse on the PED to measure its energy. The wire grid polarizer (GP), placed before the OPM3 allows to characterize the THz polarization. **c** For the electro-optic measurements, the parabolic mirror (OPM4) focuses the THz pulse into a 55 μm thick gallium phosphide (GaP) crystal. A pair of GPs reduces the THz field strength to ensure a linear response. Through a hole in OPM4 the 680 nm synchronized probe pulse focuses into the GaP crystal and then is guided to the balanced detection setup. For the cross-phase modulation experiment the GaP crystal is replaced by the 1 mm thick zinc telluride (ZnTe) crystal and the 761 nm probe pulse is guided to the spectrometer. **d** The Michelson interferometer measures the THz field autocorrelation and consists of a pellicle beam splitter (PBS) and two flat mirrors: fixed (M1) and movable (M2). At the exit, a parabolic mirror (OPM5) focuses the radiation on the PED.

In our experiments we use a powerful mid-infrared laser source, based on a hybrid OPA/OPCPA system^41^ which is capable to generate sub-100 fs laser pulses with the central wavelength of 3.9 μm, maximum pulse energy of 30 mJ at a 20 Hz repetition rate. For the THz generation we apply a standard two-color (*ω*–2*ω*) laser excitation scheme (see Fig. 1). At first, we let the fundamental laser pulse (*ω*) propagate through a quarter waveplate (QWP) and then through a 100 μm thick type I gallium selenide (GaSe) crystal with clear aperture diameter of 7 mm, in which the second harmonic pulse (2*ω*) is generated. Then, using an off-axis parabolic mirror (OPM1, 150 mm focal distance) we focus the two-color laser pulse into ambient air to produce a plasma channel. To gather the THz radiation, generated in the plasma channel, we use another parabolic mirror (OPM2, 150 mm focal distance).

Separating the generated THz radiation from the rest of the spectrum, and especially from the radiation of the fundamental 3.9 μm pulse whose central frequency, equal to 77 THz, lies quite close to the THz frequencies of interest, is not a trivial task. To address this challenge we use a combination of spatial and frequency filters. First, after the plasma channel and before the collecting mirror OPM2, we place a metallic disk (MD in Fig. 1a). This spatial filter blocks the majority of the mid-infrared radiation and generated supercontinuum (that propagate mainly along the optical axis), while the conically emitted^44,45^ and stronger diverging THz radiation passes around it. To remove the rest of the unwanted radiation, we use a longpass filter (5 mm thick high density polyethylene (HDPE) plate) which we place after the OPM2 mirror. This combination of filters was enough for the THz filtration. Nevertheless, in order to prevent the saturation of the pyroelectric detector (PED, SPI-A-62-THz Gentec-EO) by the intense THz radiation we use an extra set of longpass filters (one 5 mm HDPE plate, one 2 mm thick high resistivity float zone Silicon wafer, and one 0.5 mm thick low resistivity black Silicon wafer) in all measurements where the pyroelectric detector was involved. Information for the details on the spectral transmissivity of the filters can be found in Methods and in the Supplementary Note 1.

**Fig. 2:**
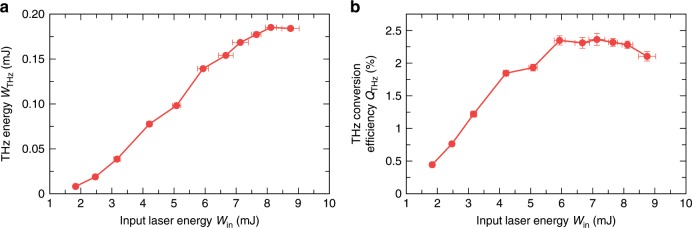
THz energy and THz conversion efficiency. Dependence of THz energy *W*_THz_ **a** and the corresponding THz conversion efficiency *Q*_THz_ **b** on the input laser energy *W*_in_. Error bars represent the standard deviation over a number of measurements.

After filtering out the THz radiation it was guided to one of our detection setups (see Fig. 1b, c, and d). To measure the energy of the generated THz pulses we focus them, using a parabolic mirror (OPM3, 100 mm focal distance), on the pyroelectric detector (see Fig. 1b). In the Supplementary Note 2 we provide a detailed description on how we restore the original values of the THz energy taking into account the calibration of the pyroelectric detector and the frequency-dependent transmission coefficients of the filters. Figure 2 shows the dependence of the restored THz energy after the filament and the corresponding THz conversion efficiency on the input laser energy *W*_in_ (calculated as the sum of the energies of the fundamental and second harmonic pulses after the GaSe crystal). We see that with the increase of the input laser energy the THz energy grows and reaches a maximum value of 0.185 mJ for *W*_in_ equal to 8.12 mJ. In turn, Fig. 2b shows that the maximum THz conversion efficiency reaches 2.36%. These values of THz energy and THz conversion efficiency greatly exceed all previously reported values obtained in experiments on two-color filamentation. For example, the THz conversion efficiency obtained in our experiment is more than two orders of magnitude higher compared to typical values reported for 0.8 μm two-color laser pulses.

It is worth noting that the power of our THz source is high enough to be measured by standard laser power meters. Indeed, using a mid-infrared laser power meter we were able detecting the generated THz pulses using only the metallic disk and one 5 mm thick HDPE filter. To verify that the measured energy comes from the THz radiation, we suppressed the THz emission either by detuning the laser compressor or the second harmonic crystal out of their optimal conditions for efficient THz generation. In both cases, the signal on the power meter disappeared. The measured THz energy was of the same order of magnitude as the one obtained using the pyroelectric detector. Yet, given that this power meter is not calibrated for the THz part of the spectrum, we decided to report here only measurements performed using our calibrated pyroelectric detector.

Finally, although THz emission through optical rectification in GaSe crystals can occur^46,47^, we have verified that under the phase matching conditions during our experiments no THz radiation was observed originating from the GaSe crystal. To confirm this, a HDPE filter was placed exactly after the GaSe crystal allowing any THz radiation produced by the crystal to pass, while the blocked laser beam did not propagate further for creating a filament. Under these conditions the signal in our pyroelectric detector dropped below its detection limits confirming that all the recorded THz radiation was produced by the filament.

**Fig. 3:**
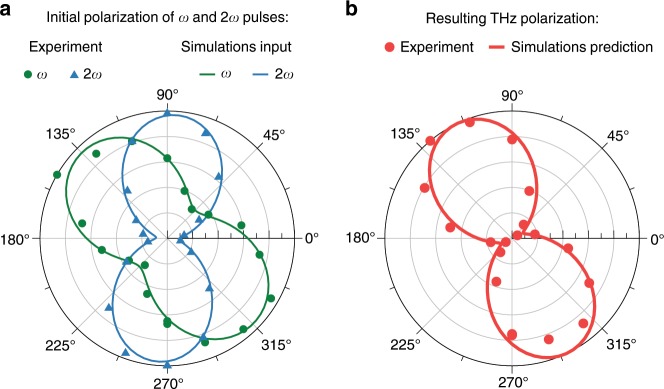
Polarizations of laser and THz pulses. **a**. Initial polarization of the fundamental (*ω*) and second harmonic (2*ω*) pulses measured in experiment (marks) and used as input in simulations (lines). **b** Resulting THz polarization measured in experiment for input laser energy of 8.75 mJ (marks) and the corresponding prediction from the simulations (line).

In order to characterize the polarization of the generated THz pulses, we use the same detection setup but with a rotating wire grid polarizer (Tydex) placed before the focusing mirror OPM3 (see GP in Fig. 1b). However, let us first mention the initial polarizations of the fundamental and second harmonic pulses. In order to maximize the THz emission we adjust the fast axis of the quarter waveplate (see QWP in Fig. 1a) to convert the polarization of the fundamental laser pulse from linear to elliptical. Then we tune the phase-matching angle of the GaSe crystal (out from the optimal angle for type I second harmonic generation, 12.1°, towards phase matching angle for type II second harmonic generation, 17.3°) to obtain the highest THz energy. As a result, at the exit of the crystal the *ω* and 2*ω* pulses become elliptically polarized as depicted in Fig. 3a, which shows that the main axes of the polarization ellipses of the *ω* and 2*ω* fields are rotated by 135° and 86°, respectively. Under these conditions we achieve a maximal mutual projection of the polarizations of the fundamental and the second harmonic beams on the same axis — the configuration being closest to the case of collinear polarizations of *ω* and 2*ω* fields, which is shown to be optimal for THz generation^48,49^. Nevertheless, despite of the elliptical polarization of the fundamental and second harmonic pulses, our measurements show that the generated THz pulses are linearly polarized and their polarization plane is rotated by 121° — in-between the major polarization axes of *ω* and 2*ω* fields (see Fig. 3b, dots).

For the spectral characterization of the generated THz pulses we apply either electro-optic sampling or field autocorrelation techniques. The electro-optic sampling technique allows one to measure the THz pulse electric field coherently. In the detection setup for the electro-optic sampling (see Fig. 1c) we use a parabolic mirror (OPM4, 50 mm focal distance) to focus the generated THz pulse into a 55 μm thick gallium phosphide (GaP) crystal. Through a hole in the mirror we send a synchronized 40 fs probe pulse centered at 680 nm (see Methods), which we also focus into the GaP crystal with a lens of 150 mm focal distance. After the crystal, we guide the probe pulse into a balanced detection setup^50^, whose readings give us a signal proportional to the THz electric field. To guarantee a linear response of the GaP crystal, we reduce the THz intensity by using, together with the metallic disk in front of the plasma, two 5 mm thick HDPE filters and a pair of wire-grid polarizers.

**Fig. 4:**
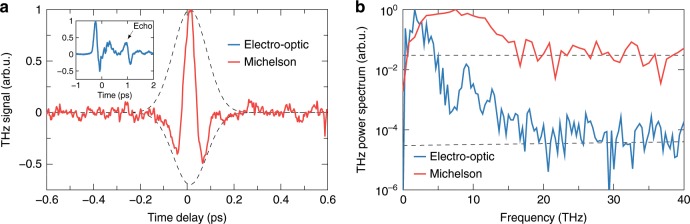
THz field and spectrum. **a** THz signals measured by electro-optic sampling (inset, blue) and by Michelson interferometer (red). **b** THz power spectra (solid lines) obtained by the above techniques together with the noise levels of each technique (dashed lines).

The inset in Fig. 4a shows the measured THz electric field (blue line) averaged over 3 consecutive scans. Figure 4b, shows the corresponding power spectrum obtained by Fourier transformation. The dashed line indicates the noise level measured under the same experimental conditions with the THz beam blocked. Because of the finite thickness of the detection crystal multiple reflections of the THz pulse occur, the first of which appears as an echo in Fig. 4a and is responsible for the Fabry-Perot resonances in the spectrum. Due to the excitation of transverse-optical (TO) lattice vibration of the detection crystal^51^, the detection bandwidth is limited mainly to the lower part of the THz spectrum (<8 THz). However, due to the small crystal thickness and the high dynamic range of the detection technique higher frequencies (up to 20 THz) are resolved by the crystal, indicating the ultra-broadband nature of our source.

An additional measurement of the spectral content of the THz pulse was obtained using field autocorrelation. Although this technique does not allow us to measure the phase of the THz pulse, it has no restriction on the detection bandwidth. The detection setup, which we use for the autocorrelation measurements is based on a Michelson interferometer (see Fig. 1d). The interferometer consists of a pellicle beam splitter (PBS) and two flat mirrors (M1 and M2). Mirror M1 is fixed while mirror M2 is placed on a motorized stage. After the PBS, we focus the interfered THz radiation, using a parabolic mirror (OPM5, 100 mm focal distance), on the same pyroelectric detector as we applied for the energy and polarization measurements using the same set of long-pass filters to protect the saturation of the detector. Figure 4a shows the recorded THz interferometric signal (red line) averaged over 5 consecutive scans.

By applying a Fourier transform to the THz interferometric signal, we obtain the power spectrum of the detected THz field shown in Fig. 4b (red line). The noise level of the measurement is indicated by the dashed line. We see that the THz spectrum peaks at around 7.5 THz and extends to higher frequencies up to about 16 THz in agreement with the electro-optic measurement. The frequencies up to 20 THz resolved by electro-optic sampling are missing from the interferometric spectrum due to the presence of the Black silicon filter that strongly attenuates this part of the spectrum (see Supplementary Fig. 1).

To estimate the amplitude of the THz electric field, we use the data on spatial and temporal shapes of the generated THz pulse (see the details in the Supplementary Note 3). Briefly, to measure the spatial profile of the THz beam in the focus of the OPM4 mirror we scan a knife-edge across the beam cross-section and measure the transmitted energy by the pyroelectric detector. For the temporal shape, we use the THz waveform recorded by the electro-optic sampling. However, since the detection bandwidth of this technique is limited, the retrieved THz pulse duration will be overestimated leading to the underestimation of the THz field amplitude. Therefore, we also estimated the THz pulse duration by fitting the THz autocorrelation signal using a common analytic formula for the temporal THz field distribution (see Supplementary Note 3). As a result, with the two temporal shapes measured by the electro-optic sampling and retrieved from Michelson interferometry, we conclude that the THz electric field amplitude at the focus of the OPM4 mirror lies in-between 100 and 150 MV cm^−1^, and the corresponding THz magnetic field amplitude in-between 33 and 50 T.

### Numerical simulations

We performed detailed numerical simulations of the two-color mid-infrared filamentation and the concomitant THz generation under the present experimental conditions, taking into account all parameters and especially the polarizations of the input laser pulses. The objective being to understand the implications of the experimental limitations, evaluate the reproducibility and thus the validity of our calculations versus the experimental findings. Finally, after the validation of our simulations we can repeat them for experimental conditions which are optimal for our laser source.

**Fig. 5:**
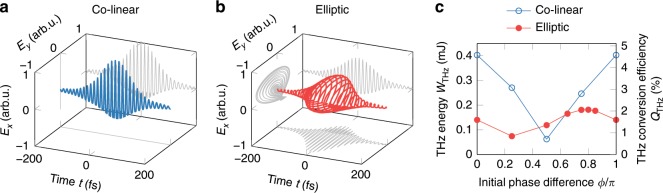
Numerical simulation. Initial two-color field with **a** collinearly and **b** elliptically polarized fundamental and second harmonic pulses. The initial phase differences *ϕ* between the *ω* and 2*ω* fields are 0 for collinear and 0.8*π* for elliptical polarization. **c** Dependence of THz energy and THz conversion efficiency on the initial phase difference *ϕ* for collinearly and elliptically polarized *ω* and 2*ω* fields.

For our simulations we use the unidirectional pulse propagation equation (UPPE)^52,53^ coupled with the rate equation for plasma concentration^54^. We present the detailed description of our numerical model and initial conditions in the Supplementary Note 4. Briefly, as the initial condition for the UPPE equation we take two-color laser pulses with two different polarization states. In the first state the polarizations of *ω* and 2*ω* fields are linear and parallel to each other. In the second state *ω* and 2*ω* fields have elliptical polarizations as the ones measured in the experiment (see Fig. 3a). For the spatial shape of the fundamental pulse we use the beam profile measured by a pyroelectric camera (Pyrocam III, Ophir-Spiricon Inc.), while for its exact temporal shape and phase we take the data obtained from the second harmonic generation FROG. In turn, to find the shape and phase of the second harmonic pulse we square the field of the fundamental pulse. Figures 5a and b show the initial two-color fields with the two mentioned polarization states. In both cases the input energy is equal to 8 mJ for the fundamental and 0.8 mJ for the second harmonic pulse (the total input laser energy is 8.8 mJ).

Unfortunately, in numerical simulations we do not know in advance what initial phase difference *ϕ* between the *ω* and 2*ω* fields gives us the highest THz yield. Therefore, we made a parametric study with different initial *ϕ* values in order to find the case with maximal THz energy. Figure 5c shows how the THz energy and THz conversion efficiency depend on the initial phase difference *ϕ* for both collinearly and elliptically polarized two-color fields. We see that the maximum energy of the generated THz radiation is reached at *ϕ* = 0 for collinearly and at *ϕ* = 0.8*π* for elliptically polarized *ω* and 2*ω* fields. Note that these phase differences correspond only to the initial moment, where there is no plasma yet; during further propagation they will change due to nonlinear phase accumulation. In the following discussion we present the results obtained with the above values of *ϕ*.

The careful incorporation of initial conditions together with our rigorous propagation model allow us to give accurate predictions, which are in excellent quantitative agreement with the experimental data. For instance, in Fig. 3b one can see that our simulations with elliptically polarized *ω* and 2*ω* pulses predict exactly the measured THz polarization. Thus, now we can use our simulations to estimate how a change of the initial laser polarization will be affecting the THz emission in our experiments. In our simulations the values of the maximum THz energy for the collinear and elliptical polarizations are, respectively, 0.403 and 0.181 mJ. That is, in the case of collinear polarizations the energy of the generated THz pulses is 2.23 times higher compared to the case of elliptical polarizations. Using this factor we can estimate what will be the THz energy and THz conversion efficiency in our experiment if one would be able to properly align the polarizations of the fundamental and second harmonic pulses. Taking into account that for the elliptically polarized initial laser pulses we experimentally measured 0.185 mJ of THz energy, we can expect that in case of collinearly polarized *ω* and 2*ω* fields the THz energy will increase up to 0.413 mJ, which will correspond to  ~4.7% of THz conversion efficiency. This THz conversion efficiency is very close to the one predicted in our previous theoretical studies on two-color filamentation of 3.9 μm laser pulses^37,38^. Under these conditions the corresponding peak THz electric and magnetic fields are expected to exceed the values of 200 MV cm^−1^ and 66 T respectively.

### Strong THz field application

To illustrate the strength of the THz fields generated by the mid-infrared filaments, we conducted a pump-probe experiment on nonlinear cross-phase modulation (XPM) of a THz pump and a visible probe pulse inside a 1 mm zinc telluride (ZnTe) crystal. Previously, similar XPM experiments were reported by Shen et al.^55,56^ in ZnTe and by Vicario et al.^57^ in GaP crystals.

For our XPM experiment we adopt the electro-optic detection setup (see Fig. 1c). This time, as a probe pulse we take the radiation generated in the vicinity of the fifth harmonic of the mid-IR pulses propagating in air and centered at 761 nm. The probe pulse is focused into the ZnTe crystal and properly synchronized with the THz pulse. After the crystal, we collect the probe pulse and send it to a spectrometer. In order to maximize the THz energy that enters the nonlinear crystal we reduced the number of longpass filters by leaving only a single 5 mm thick HDPE plate.

**Fig. 6:**
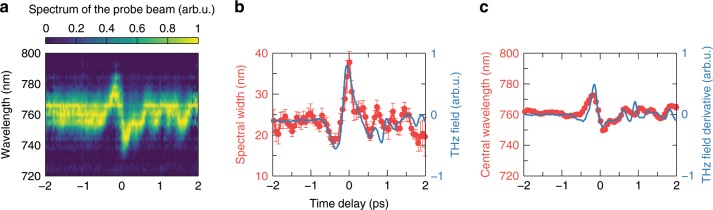
THz field induced cross phase modulation. **a** Spectrum of the probe beam as a function of the time delay between the THz and probe pulses. Spectral width **b** and central wavelength **c** of the probe pulse (red dot-lines) together with the THz field and the first time derivative of the THz field, respectively, (blue lines) as a function of the time delay between the THz and probe pulses. Error bars represent the standard deviation.

 Figure 6a shows the spectrum of the probe pulse as a function of the relative time delay between the probe and the THz pulse. One can see that, when the THz and probe pulses overlap in time, the spectrum of the probe pulse becomes strongly modulated: we observe a shift of the central wavelength as well as a change of the spectral width. In particular, Fig. 6b shows how the spectral width of the probe pulse (measured as the FWHM of the spectrum) depends on the time delay between the probe and THz pulses. In the same figure we plot the THz field measured by electro-optic sampling using the same 1 mm ZnTe crystal. As we can see there is a clear correlation between the THz field amplitude and the variations of the spectral width of the probe pulse. In turn, Fig. 6c shows that the central frequency of the probe pulse initially undergoes a red shift which then gives way to a blue shift, both about 12 nm. These shifts are proportional to the first derivative of the THz field^55,57^ (see the blue line in Fig. 6c). Error bars in Fig. 6c lie within the red dot points.

## Discussion

In conclusion, we have demonstrated an extremely efficient generation of high power THz pulses from mid-infrared two-color laser filaments. In our experiments we obtained single cycle THz pulses with energies up to 0.185 mJ, THz conversion efficiencies up to 2.36% and THz field amplitudes exceeding 100 MV cm^−1^. Such a level of THz yield makes mid-infrared two-color filaments the most efficient source of THz radiation among existing plasma-based THz sources. The THz conversion efficiency demonstrated in our experiment is more than two orders of magnitude higher compared to typical values, which can be obtained with two-color pulses from Ti:sapphire lasers.

The present work provides a realistic solution to sources of extreme strength THz fields. Even though our experimentally recorded THz power was limited by technical restrictions, like for instance the use of spectral filters that absorb a part of the radiation, the control of the polarization of the driving laser and its second harmonic, and the energy losses in the second harmonic nonlinear crystal (limiting the maximum usable energy of our two-color laser pulses at 8.75 mJ), the outlook looks bright. The deep understanding of the physics of these sources, demonstrated here by the excellent agreement between simulations and experimental findings, allows for identifying ways to further improving these sources. Indeed, our simulations show^38^ that by merely increasing the input laser pump energies in the range of a few tens of millijoules, the energy of produced THz pulses will increase to the multi-millijoule level, and their peak electric and magnetic fields to the gigavolt per centimeter and kilotesla level, respectively.

Quasi-static ultrashort electric and magnetic bursts at these intensities will enable free space extreme nonlinear and relativistic science. Moreover, as these sources are produced by affordable tabletop laser systems, operating at reasonably high repetition rates, beyond of their uniqueness, they can find useful applications, like for instance in charged particle accelerators.

## Methods

### Electro-optic sampling

To characterize the electric field of the generated THz pulses, we apply the detection setup (see Fig. 1c) based on electro-optic sampling technique. To generate the probe beam for this setup we use a home-built, white-light seeded, non-collinearly pumped optical parametric amplifier (NOPA) based on type I BBO crystal. The synchronization between the OPCPA system and the NOPA is achieved by a common Yb:CaF2 pump source, which at 500 Hz repetition rate drives both, OPA stages of the 3.9 μm OPCPA system^41^ and, after frequency doubling, the NOPA. In order to reduce the repetition rate of the NOPA to 20 Hz, in the white-light generation stage we installed an electro-optical switch. After, using a pair of SF10 prisms, we compressed the output pulses of NOPA (centered at 680 nm) down to 40 fs.

### Longpass filters characterization

We characterized the frequency-dependent transmission coefficients of the longpass filters used in the experiments in the range from 1.1 to 200 THz using a Fourier-transform infrared vacuum spectrometer (BRUKER Vertex 70v). Supplementary Fig. 1 shows the separate transmission coefficients of each filter as well as the total transmission coefficient of the full filter set. After the filers, the transmittance of the THz radiation with frequencies below 20 THz is by several orders of magnitude higher compared to the rest of the spectrum.

## Supplementary information

Supplementary Information

## Data Availability

All relevant data are available from the authors.
