# Supplementary material for: Observation of extremely efficient terahertz generation from mid-infrared two-color laser filaments
Source: Nat Commun. 2020 Jan 15;11:292. doi: 10.1038/s41467-019-14206-x (PMC6962375; doi:10.1038/s41467-019-14206-x)
Supplement: Supplementary file 1 — Supplementary Information [file 41467_2019_14206_MOESM1_ESM.pdf]

Supplementary Information for

"Observation of extremely efficient terahertz generation from mid-infrared two-color laser filaments"

Koulouklidis et al.

Supplementary Note 1: Longpass filters

In our experiments, we use a set of longpass filters to separate the generated THz radiation from the rest of the spectrum as well as to prevent the saturation of the pyroelectric detector from the high THz intensity. The set of filters includes two 5 mm thick high density polyethylene (HDPE) plates, one 2 mm thick high resistivity float zone Silicon (HRFZ-Si) wafer, and one 0.5 mm thick low resistivity black Silicon (Black-LR-Si) wafer. Supplementary Figure 1 shows the separate transmission coefficients of each filter as well as the total transmission coefficient of the full filter set.

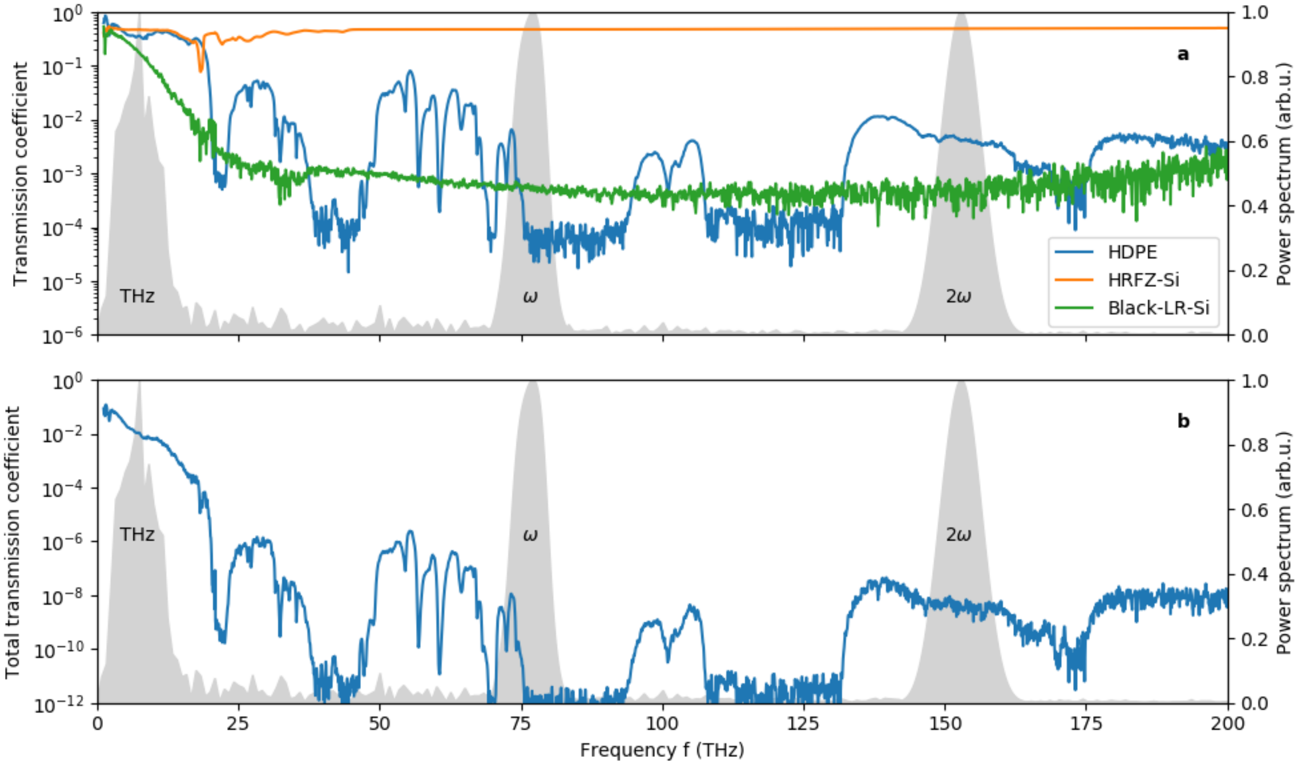

Supplementary Figure 1. Transmission coefficients of the long pass filters used in the experiment. **a** Transmission coefficients of HDPE, HRFZ-Si, and Black-LR-Si filters. **b** Total transmission coefficient of the full filter set (two HDPE, one HRFZ-Si, and one Black-LR-Si filters). Gray shaded plots show the initial power spectra of the fundamental (ω) and second harmonic (2ω) pulses, as well as the power spectrum of the generated THz pulse.

Supplementary Note 2: Retrieval of the THz energy

In the experiment, to measure the energy of the THz pulses, we use the pyroelectric detector (PED). In order to translate the readings of the PED to absolute energy units (joules) we use the following approach. First, we note that the signal $S(\tau)$ measured by the Michelson interferometer is proportional to the autocorrelation function of the THz electric field $E(t)$:

$$S(\tau) \propto \int_{-\infty}^{\infty} E(t)E(t+\tau)\mathrm{d}t. \tag{1}$$

According to the Wiener-Khinchin theorem the amplitude spectrum of $S(\tau)$ is proportional to the power spectrum of $E(t)$:

$$\widetilde{S}(f) \propto \left|\widetilde{E}(f)\right|^2, \tag{2}$$

where $\widetilde{S}(f)$ and $\widetilde{E}(f)$ are the amplitude spectra of the interferometric signal and THz electric field, respectively. Then, using the Plancherel's theorem, which states that the integral of the squared modulus of a function is equal to the integral of the squared modulus of its spectrum (i.e. power spectrum), we can find the energy W_{THz} of the THz pulse:

$$\int_{-\infty}^{\infty} \widetilde{S}(f)\mathrm{d}f \propto \int_{-\infty}^{\infty} \left|\widetilde{E}(f)\right|^2 \mathrm{d}f = \int_{-\infty}^{\infty} |E(t)|^2 \mathrm{d}t \propto W_{\mathrm{THz}}. \tag{3}$$

In turn, to find the proportionality coefficient we use the data on the calibrated PED response function and transmission coefficients of the filters.

The blue lines in Supplementary Figure 2 show the THz signal $S(\tau)$ measured by the Michelson interferometer and the corresponding THz spectrum $\widetilde{S}(f)$. According to our approach, in order to find the energy of the THz pulse we have to integrate the spectrum $\widetilde{S}(f)$ over all frequencies (see Supplementary Equation (3)). However, after passing through the filters, high frequency spectral components of the THz spectrum were suppressed and their amplitudes dropped down below the noise level. Thus, starting from some frequency, all spectral amplitudes at higher frequencies represent just noise and should be excluded from the calculation of the THz energy. In our analysis we empirically choose the cut-off frequency f_{cut}:

$$f_{\mathrm{cut}} = 15.5\ \mathrm{THz}, \tag{4}$$

and assume that all spectral components of the THz spectrum located above f_{cut} are irretrievably lost and can not be reconstructed. In order to avoid the impact of noise on the THz energy calculations, we set all spectral amplitudes of the THz spectrum $\widetilde{S}(f)$ at frequencies above f_{cut} to zero. The orange lines in Supplementary Figure 2 show the THz spectrum and the corresponding THz signal obtained after this spectral restriction procedure. In all our further analysis we will use this restricted THz spectrum.

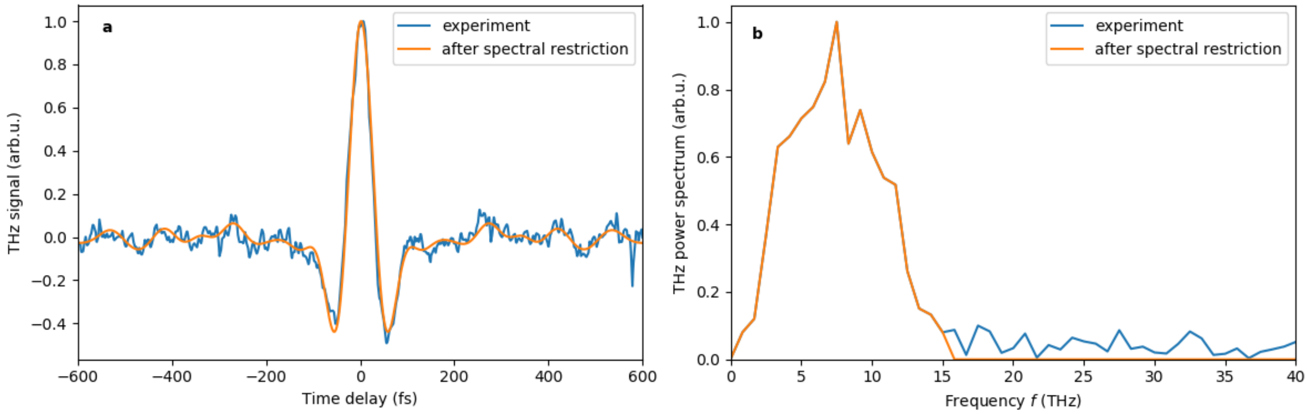

Supplementary Figure 2. THz signal and its spectrum. **a** THz signal measured by Michelson interferometer. **b** The corresponding THz power spectrum before (blue line) and after (orange line) the spectral restriction procedure.

During the energy measurements, after the THz pulses pass through the set of filters they are absorbed by the PED, wherein an electric current is generated. We use the voltage V_{p} of this current to measure the THz pulse energy W_{THz}. When the response of the PED is linear (we use the additional filters in the filter set specially to maintain this detection regime), V_{p} is proportional to W_{THz}. In turn, since according to Supplementary Equation (3) the THz energy W_{THz} is proportional to the integrated spectrum $\widetilde{S}(f)$ of the THz interferometric signal, we can write

$$V_{\mathrm{p}} = C_{\mathrm{v}} \int_{-\infty}^{\infty} \widetilde{S}(f) \mathrm{d}f, \tag{5}$$

where C_{v} is the coefficient of proportionality, which we can rewrite as

$$C_{\mathrm{v}} = \frac{V_{\mathrm{p}}}{\int_{-\infty}^{\infty} \widetilde{S}(f) \mathrm{d}f}. \tag{6}$$

Using this coefficient we can renormalize the THz spectrum:

$$\widetilde{S}_{\mathrm{n}}(f) = C_{\mathrm{v}} \widetilde{S}(f). \tag{7}$$

By integrating the renormalized spectrum $\widetilde{S}_{\mathrm{n}}(f)$ we obtain

$$\int_{-\infty}^{\infty} \widetilde{S}_{\mathrm{n}}(f) \mathrm{d}f = \int_{-\infty}^{\infty} C_{\mathrm{v}} \widetilde{S}(f) \mathrm{d}f = V_{\mathrm{p}}. \tag{8}$$

Therefore, since frequencies f are measured in Hz and V_{p} in volts, the units of $\widetilde{S}_{\mathrm{n}}(f)$ are V/Hz. In other words, $\widetilde{S}_{\mathrm{n}}(f)$ tells us how many volts of the resulting PED signal are produced by each spectral component.

Next, we have to calibrate the response of the PED. The manufacturer (Gentec-EO) of the PED provides the wavelength-dependent response function $F_{\mathrm{p}}(f)$ in the wavelength range from 0.25 to 395 μm. This function describes the relative sensitivity of the PED at different frequencies. To calibrate the PED we irradiated it with laser pulses at 1 μm central wavelength and recorded the PED voltage readings for several laser pulse energies. Then, after the least square fitting of the obtained data, we found the proportionality coefficient $C_{\mathrm{p}} = 0.1272 \times 10^{-6}$ J/V which relates the PED readings in volts to the energy of the incident pulses in joules. Next, we renormalized the response function $F_{\mathrm{p}}(f)$ by dividing $F_{\mathrm{p}}(f)$ by its value at 1 μm and then multiplying by the coefficient C_{p}. With the renormalized response function $F_{\mathrm{p}}(f)$ we mapped the spectral amplitudes of $\widetilde{S}_{\mathrm{n}}(f)$ spectrum from V/Hz to J/Hz:

$$\widetilde{S}_{\mathrm{np}}(f) = F_{\mathrm{p}}(f)\widetilde{S}_{\mathrm{n}}(f). \tag{9}$$

The obtained spectrum $\widetilde{S}_{\mathrm{np}}(f)$ is measured in J/Hz units and shows how much THz energy is contained at each specific frequency. Supplementary Figure 3 shows the renormalized PED response function $F_{\mathrm{p}}(f)$ together with the resulting THz spectrum $\widetilde{S}_{\mathrm{np}}(f)$.

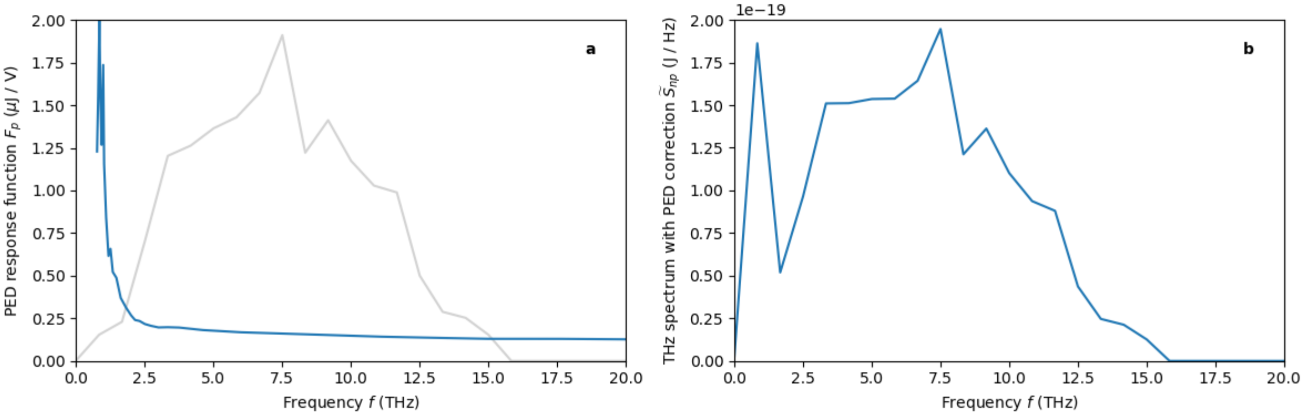

Supplementary Figure 3. Calibration of the THz spectrum with the response function of the pyroelectric detector (PED). **a** Dependence of the calibrated PED response function F_{p} on frequency f. The gray line shows the spectrum of THz signal measured by Michelson interferometer. **b** Dependence of the THz spectrum $\widetilde{S}_{\mathrm{np}}$, after PED correction, on frequency f.

As a final step, in order to find the spectrum of THz pulses before the filters, we divide the THz spectrum $\widetilde{S}_{\mathrm{np}}(f)$ by the total frequency-dependent transmission coefficient $T_{\mathrm{tot}}(f)$ of the filter set (see Supplementary Figure 1b):

$$\widetilde{S}_{\mathrm{npf}}(f) = \widetilde{S}_{\mathrm{np}}(f)\,/\,T_{\mathrm{tot}}(f). \tag{10}$$

Since $T_{\mathrm{tot}}(f)$ is dimensionless, the units of the resulting spectrum $\widetilde{S}_{\mathrm{npf}}(f)$ remain the same, that is, J/Hz. Supplementary Figure 4 shows the THz spectrum $\widetilde{S}_{\mathrm{npf}}$ and the corresponding THz signal together with the original THz signal obtained without PED and filters corrections.

Finally, to find the THz energy W_{THz} we integrate the spectrum $\widetilde{S}_{\mathrm{npf}}$ over all frequencies (see Supplementary Equation (3)):

$$W_{\mathrm{THz}} = \int_{-\infty}^{\infty} \widetilde{S}_{\mathrm{npf}}(f)\mathrm{d}f. \tag{11}$$

Thus, the above procedure allows us to map the voltage V_{p} of the PED to the energy W_{THz} of the generated THz pulses. For example, for initial laser pulse energy equal to 8.12 mJ we obtained $V_{\mathrm{p}} = 8.91$ V which results in a THz energy $W_{\mathrm{THz}} = 0.185$ mJ and corresponding THz conversion efficiency equal to 2.28%.

Supplementary Note 3: Retrieval of the THz electric field amplitude

With the energy of the THz pulse we can retrieve the amplitude of the THz electric field. In general, energy W can be represented as

$$W = \iiint_{-\infty}^{\infty} I(x,y,t)\,\mathrm{d}x\,\mathrm{d}y\,\mathrm{d}t, \tag{12}$$

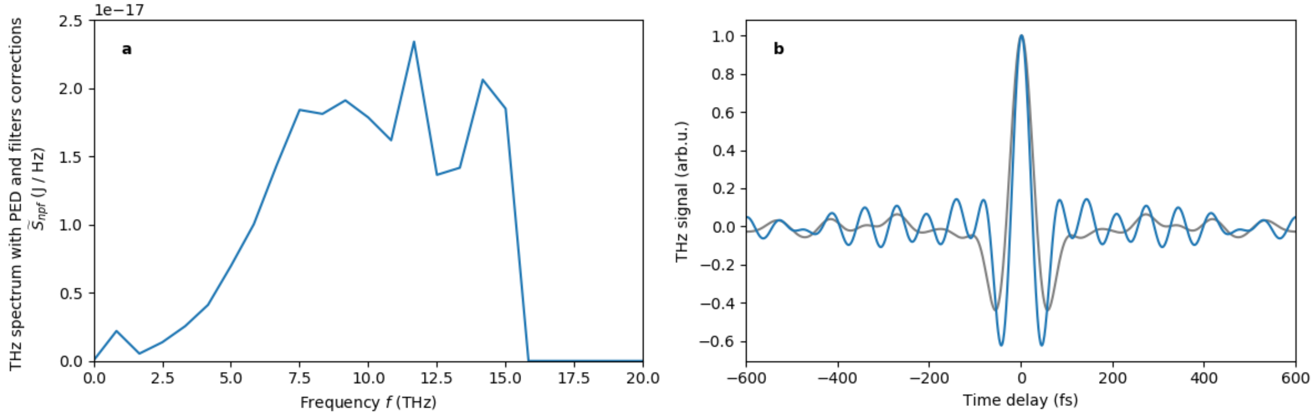

Supplementary Figure 4. THz signal and its spectrum after correction for the filters and response of the pyroelectric detector (PED). **a** THz spectrum $\widetilde{S}_{\rm npf}$, obtained after PED and filter corrections, as a function of frequency f. **b** The corresponding THz signal as a function of time delay together with the original THz signal (gray line) obtained without the PED and filter corrections.

where $I(x,y,t)$ is the pulse intensity given by

$$I(x,y,t) = \frac{1}{2} n_0 \varepsilon_0 c_0 \, |E(x,y,t)|^2, \tag{13}$$

where n_0 is the medium refractive index at the pulse central frequency, ε_0 is the vacuum permittivity, c_0 is the speed of light in vacuum, and $E(x,y,t)$ is the pulse electric field. We assume that the electric field $E(x,y,t)$ can be written as

$$E(x,y,t) = A \, E_{\rm xy}(x,y) \, E_{\rm t}(t), \tag{14}$$

where A is the field amplitude while $E_{\rm xy}(x,y)$ and $E_{\rm t}(t)$ represent the normalized spatial and temporal field profiles. Thus, Supplementary Equations (12)–(14) allow us to write the field amplitude A as

$$A = \sqrt{\frac{2}{n_0 \varepsilon_0 c_0} \frac{W}{G_{\rm xy} \, G_{\rm t}}} \tag{15}$$

where the integrated spatial and temporal profiles, $G_{\rm xy}$ and $G_{\rm t}$, are given by

$$G_{\rm xy} = \iint_{-\infty}^{\infty} |E_{\rm xy}(x,y)|^2 \, {\rm d}x \, {\rm d}y, \quad G_{\rm t} = \int_{-\infty}^{\infty} |E_{\rm t}(t)|^2 \, {\rm d}t. \tag{16}$$

To find $G_{\rm xy}$ we applied the knife-edge technique where we record the signal at the pyroelectric detector during knife-edge scan of the THz beam in the focus of the OPM4 mirror. Blue dots in Supplementary Figure 5a show the obtained data. Assuming that the THz beam has a Gaussian profile, the signal P of the pyroelectric detector will be[1]

$$P(x) = P_0 + \frac{P_{\rm max}}{2} \left[1 + {\rm erf}\left(\frac{x - x_0}{a} \right) \right], \tag{17}$$

where x is the coordinate along the direction of the knife-edge scan, P_0 and $P_{\rm max}$ are the background and maximum values of the pyroelectric signal, while erf is the error function with x_0 being the location of the THz beam maximum along x, and a is the $1/e$ THz beam radius. Using Supplementary Equation (17) we fitted the experimental data (see orange line in Supplementary Figure 5a) and obtained the THz beam radius a equal to 59.3 μm. Taking into account this value of a and our assumption about the Gaussian shape of the THz beam, we can calculate the integrated spatial profile $G_{\rm xy}$ simply as $G_{\rm xy} = \pi a^2$.

For the integrated temporal profile $G_{\rm t}$, we take the time integral of the THz waveform recorded by the electro-optic sampling technique (see the inset of Fig. 4a in main text). Since the detection bandwidth of this technique is limited, the strength of the

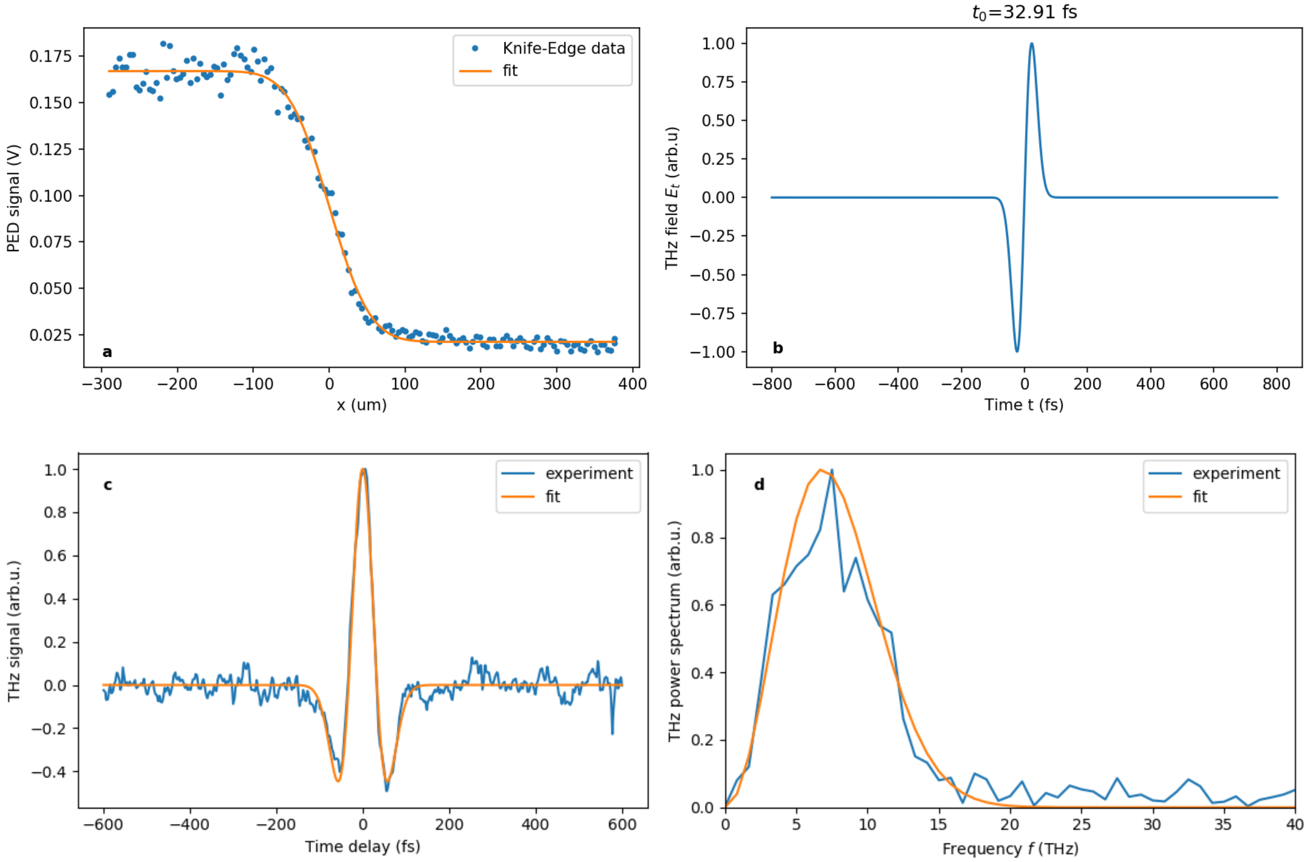

Supplementary Figure 5. Estimations of the spatial and temporal shapes of the THz pulse. **a** The profile of the THz beam at the focus of OAP4 mirror measured by knife-edge technique (blue dots) together with its fit calculated with Supplementary Equation (17) (orange line). **b** The model THz electric field used for the fitting of the THz temporal profile. **c** The THz signal measured by the Michelson interferometer together with the best-fit autocorrelation calculated for the model THz electric field. **d** The comparison of the corresponding THz spectra.

THz field is underestimated. Therefore we also used the THz signal recorded with the Michelson interferometer. We applied a nonlinear curve fitting in order to fit this THz signal with the autocorrelation result of the following THz temporal profile[2]:

$$E_{\mathrm{t}}(t) = E_0 \frac{t}{t_0} \exp\left(-\frac{t^2}{t_0^2}\right), \tag{18}$$

where E_0 is the amplitude normalizing E_{t} to unity and t_0 is the fitting parameter. In Supplementary Figure 5b we can see how the $E_{\mathrm{t}}(t)$ profile looks like. As the result of the fitting procedure we obtain $t_0 = 32.91$ fs. Supplementary Figures 5c and d show that, with the chosen THz temporal profile and obtained t_0 value, we can fit the measured THz interferometric signal accurately both in the temporal and spectral domains. For this case, G_{t} is the time integral of $|E_{\mathrm{t}}(t)|^2$ with $E_{\mathrm{t}}(t)$ given by Supplementary Equation (18) and $t_0 = 32.91$ fs.

As a result, we have an upper and a lower limit for G_{t}, given by the electro-optic sampling and Michelson interferometer, respectively. By substituting these values of G_{t} together with the calculated value of G_{xy} into Supplementary Equation (15), we found that the electric field of the THz pulse with the previously retrieved energy of 0.185 mJ, has an amplitude between 100 and 150 MV cm^{-1} which corresponds to the THz magnetic field amplitudes in-between 33 and 50 T (see Supplementary Table 1).

Supplementary Note 4: Numerical simulation

In our studies we numerically simulate the nonlinear propagation of powerful femtosecond laser pulses in atmospheric air. In these studies the polarization of the laser pulses can differ from the linear one. In order to describe the propagation of a laser

Supplementary Table 1. Lower and upper limits of the THz electric and magnetic field amplitudes obtained by electro-optic sampling and Michelson interferometry

	Peak THz electric field (MV cm^{-1})	Peak THz magnetic field (T)
Electro-optic	100	33
Michelson	150	50

pulse with an arbitrary polarization state we express the electric field vector $\mathbf{E}$ through a sum of its projections, E_x and E_y, on axes x and y: $\mathbf{E} = E_\text{x}\mathbf{x} + E_\text{y}\mathbf{y}$, where $\mathbf{x}$ and $\mathbf{y}$ are the corresponding unit vectors. For each of the electric field components E_i ($i = x, y$) we numerically solve the Unidirectional Pulse Propagation Equation (UPPE), given by[3–5]:

$$\frac{\partial \hat{E}_i}{\partial z} = \mathrm{i}k_\text{z}\hat{E}_i + \mathrm{i}\frac{\mu_0\mu\omega^2}{2k_\text{z}}\hat{N}_i, \qquad i = x, y, \tag{19}$$

where "i" stands for the imaginary unit, $\hat{E}_i(k_\text{x}, k_\text{y}, \omega, z)$ is the spatio-temporal spectrum of a given component E_i, $\hat{N}_i(k_\text{x}, k_\text{y}, \omega, z)$ represents the nonlinear response of the medium, $k_\text{z}(k_\text{x}, k_\text{y}, \omega) = [k^2(\omega) - k_\text{x}^2 - k_\text{y}^2]^{1/2}$ is the propagation constant, k_x, k_y, and ω are the spatial and temporal angular frequencies, $k(\omega) = n(\omega)\omega/c_0$ is the wave number with $n(\omega)$ being the frequency-dependent refractive index and c_0 is the speed of light in vacuum, while μ_0 and μ are the vacuum and medium permeabilities, respectively. In our model, the nonlinear response takes into account the third order nonlinear polarizations due to Kerr, $\mathbf{P}_\text{K}$, and Raman, $\mathbf{P}_\text{R}$, effects, the current of free electrons $\mathbf{J}_\text{F}$, and the current that is responsible for ionization losses $\mathbf{J}_\text{A}$:

$$\hat{N}_i = \hat{P}_{\text{K}i} + \hat{P}_{\text{R}i} + \frac{\mathrm{i}}{\omega}(\hat{J}_{\text{F}i} + \hat{J}_{\text{A}i}), \qquad i = x, y \tag{20}$$

with

$$\hat{P}_{\text{K}i} = (1-g)\varepsilon_0\chi^3\,\widehat{F}_{\text{K}i}, \qquad F_{\text{K}i} = \left[E_i^2 + E_j^2\right]E_i, \tag{21}$$

$$\hat{P}_{\text{R}i} = g\varepsilon_0\chi^3\,\widehat{F}_{\text{R}i}, \qquad F_{\text{R}i} = E_i\int_{-\infty}^{\infty} H(t-\tau)\left[E_i^2(\tau) + \frac{1}{3}E_j^2(\tau)\right]\mathrm{d}\tau + E_j\int_{-\infty}^{\infty} H(t-\tau)\left[\frac{2}{3}E_i(\tau)E_j(\tau)\right]\mathrm{d}\tau, \tag{22}$$

$$\hat{J}_{\text{F}i} = \frac{q_e^2}{m_e}\frac{\nu_\text{c} + \mathrm{i}\omega}{\nu_\text{c}^2 + \omega^2}\widehat{F}_{\text{F}i}, \qquad F_{\text{F}i} = \rho\left[E_i^2 + E_j^2\right]^{1/2}, \tag{23}$$

$$\hat{J}_{\text{A}i} = K\hbar\omega_0\widehat{F}_{\text{A}i}, \qquad F_{\text{A}i} = \frac{\partial\rho}{\partial t}\left[E_i^2 + E_j^2\right]^{-1/2}, \tag{24}$$

$$i, j = x, y \quad j \neq i.$$

Here $\widehat{}$ denotes the spatio-temporal spectrum, $g \in [0, 1]$ is the fraction of Raman contribution to the nonlinear polarization, $H(t)$ is the Raman response function, ε_0 is the vacuum permittivity, $\chi^3 = 4n_0^2\varepsilon_0 c_0 n_2/3$ is the cubic susceptibility with n_2 being the nonlinear index, n_0 is the medium refractive index at the central frequency of the fundamental pulse ω_0, q_e and m_e are the charge and mass of the electron, ν_c is the collisions frequency, ρ is the concentration of free electrons (in 1/m^3), and K is the order of the multiphoton ionization. The real part of $\hat{J}_\text{F}$ describes inverse Bremsstrahlung, and the imaginary part is responsible for plasma defocusing.

In our simulations we use the following medium parameters: collisions frequency[6] $\nu_\text{c} = 5 \times 10^{12}$ 1/s, nonlinear index[7] $n_2 = 3.8 \times 10^{-23}$ m^2/W (the corresponding critical power at 3.9 μm is equal to 60 GW), the fraction of Raman contribution[7] $g = 0.79$, the Raman response function[8] $H(t) = \text{step}(t)\Omega^2\exp(-\Gamma t/2)\sin(\Lambda t)/\Lambda$ with $\Lambda = (\Omega^2 - \Gamma^2/4)^{1/2}$, $\Omega = 20.6$ THz, and $\Gamma = 26$ THz.

For the complex frequency-dependent refractive index of air $n = n' + \mathrm{i}n''$ we use the following model[9]: $n(\omega) = n_\text{Peck}(\omega) + n_\text{HITRAN}(\omega)$. The real valued refractive index $n_\text{Peck}(\omega)$ is given in[10], while to calculate the complex refractive index $n_\text{HITRAN} = n'_\text{HITRAN} + \mathrm{i}n''_\text{HITRAN}$ we use the data on spectral lines of atmospheric gases from the HITRAN database[11]. The imaginary part n''_HITRAN is recalculated from the absorption coefficient given in the database, then the real part n'_HITRAN is restored using the Kramers-Kronig relations. To obtain the frequency-dependent absorption coefficient from the HITRAN database we have to specify the gas content of air. To build a realistic dispersion model of atmospheric air we assume that air consists of nitrogen N_2, oxygen O_2, CO_2, and water vapor H_2O gases. Using the experimentally measured air temperature (23.55°C), relative humidity (53%) and concentration of CO_2 gas (480 ppm) we find that the atmospheric gases have the following fractions: 77.762% of N_2, 20.671% of O_2, 1.519% of H_2O, and 0.048% of CO_2. We use these fractions as an input data for the HITRAN database to calculate the frequency-dependent absorption coefficient of air, specific for our conditions.

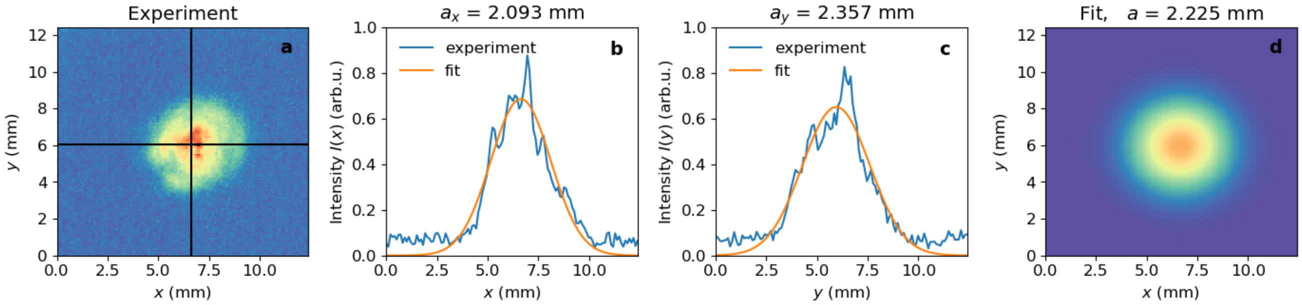

Supplementary Figure 6. The beam profile used in the initial condition for the simulations. **a** Measured initial beam profile. **b** and **c** are the cross-sections along the y and x axes, respectively (marked by the black lines on the measured profile). (d) Reconstructed Gaussian beam profile whose radius is calculated through averaging of the radii obtained by fitting the cross-sections.

As the initial condition for the UPPE (see Supplementary Equation (19)) we take a two-color laser field with spatial and temporal shapes based on experimental measurements. To find the spatial shape of the fundamental beam we measured its profile using the pyroelectric camera (Pyrocam III, Ophir-Spiricon Inc.) (see Supplementary Figure 6a). Then we took cross-sections along the x and y axes that pass through the beam's center of mass (see the black lines in Supplementary Figure 6a). We fitted these cross-sections by Gaussian functions and found the two corresponding radii (see Supplementary Figures 6b and c). The average of these two radii gave us the resulting $1/e$ radius $a_\omega = 2.225$ mm. Using this radius we obtained the Gaussian profile of the initial fundamental beam (see Supplementary Figure 6d).

To find the temporal shape and phase of the fundamental pulse we conducted FROG measurements based on second harmonic generation. Supplementary Figure 7 shows the reconstructed electric field and its power spectrum together with their phases. For our further analysis we converted (using the Hilbert transform) the real-valued electric field function into a complex-valued analytic signal E_t^ω. The real part of E_t^ω is equal to the original field function shown in Supplementary Figure 7. The modulus $|E_t^\omega|$ gives us the field envelope, while $|E_t^\omega|^2$ is proportional to the intensity of the fundamental pulse. Using $|E_t^\omega|^2$ function we found that the duration of the fundamental pulse is equal to 101 fs (FWHM).

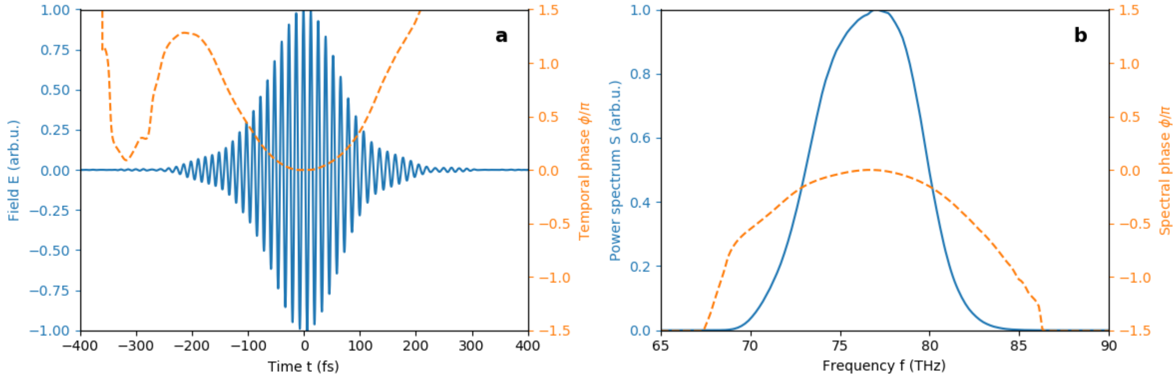

Supplementary Figure 7. The temporal pulse shape used in the initial condition for the simulations. **a** Initial temporal profile of the fundamental pulse. **b** The corresponding spectrum reconstructed from the FROG measurements.

In turn, to obtain the initial spatial and temporal profiles of the second harmonic pulse we take into account that during the second harmonic generation process the field of the second harmonic is proportional to the square of the fundamental field. Therefore, to find the initial shape of the second harmonic pulse we square the spatial and temporal profiles of the fundamental pulse. In particular, we find that the initial radius of the second harmonic beam is $a_{2\omega} = a_\omega/\sqrt{2}$.

The projections on x and y axes of the fundamental and second harmonic pulses with arbitrary polarizations can be written

as

$$E_{\mathrm{x}}^{\omega} = A^{\omega} \exp\left(-\frac{r^2}{2a_{\omega}^2}\right) \mathrm{Re}\{E_{\mathrm{t}}^{\omega}\}, \qquad E_{\mathrm{y}}^{\omega} = \varepsilon_{\omega} A^{\omega} \exp\left(-\frac{r^2}{2a_{\omega}^2}\right) \mathrm{Im}\{E_{\mathrm{t}}^{\omega}\}, \tag{25}$$

$$E_{\mathrm{x}}^{2\omega} = A^{2\omega} \exp\left(-\frac{r^2}{2a_{2\omega}^2}\right) \mathrm{Re}\left\{E_{\mathrm{t}}^{2\omega}\right\}, \qquad E_{\mathrm{y}}^{2\omega} = \varepsilon_{2\omega} A^{2\omega} \exp\left(-\frac{r^2}{2a_{2\omega}^2}\right) \mathrm{Im}\left\{E_{\mathrm{t}}^{2\omega}\right\}, \tag{26}$$

where Re{} and Im{} denote the real and imaginary parts, A^{ω} and $A^{2\omega}$ are the initial amplitudes, while $E_{\mathrm{t}}^{2\omega} = (E_{\mathrm{t}}^{\omega})^2 e^{-\mathrm{i}\phi}$ with ϕ being the initial phase difference between the ω and 2ω fields. The parameters ε_{ω} and $\varepsilon_{2\omega}$ describe the polarization state of the fundamental and second harmonic pulses. For example, ε_{ω} equal to 0 and 1 define the linear and circular polarizations of the fundamental pulse, while the intermediate values correspond to the elliptical polarization; the sign of ε_{ω} allows us to switch between the left- and right-handed polarizations.

In order to describe the orientation of the polarization ellipse in the xy plane we introduce the angles θ_{ω} and $\theta_{2\omega}$:

$$E_{\mathrm{x}}^{\omega\prime} = E_{\mathrm{x}}^{\omega} \cos\theta_{\omega} - E_{\mathrm{y}}^{\omega} \sin\theta_{\omega}, \qquad E_{\mathrm{y}}^{\omega\prime} = E_{\mathrm{x}}^{\omega} \sin\theta_{\omega} + E_{\mathrm{y}}^{\omega} \cos\theta_{\omega}, \tag{27}$$

$$E_{\mathrm{x}}^{2\omega\prime} = E_{\mathrm{x}}^{2\omega} \cos\theta_{2\omega} - E_{\mathrm{y}}^{2\omega} \sin\theta_{2\omega}, \qquad E_{\mathrm{y}}^{2\omega\prime} = E_{\mathrm{x}}^{2\omega} \sin\theta_{2\omega} + E_{\mathrm{y}}^{2\omega} \cos\theta_{2\omega}. \tag{28}$$

These angles define how the polarization ellipses of ω and 2ω fields are inclined in the input plane.

Finally, the projections on x and y axes of the total initial two-color field are given by the sum of the corresponding projections of ω and 2ω fields:

$$E_{\mathrm{x}} = E_{\mathrm{x}}^{\omega} + E_{\mathrm{x}}^{2\omega}, \qquad E_{\mathrm{y}} = E_{\mathrm{y}}^{\omega} + E_{\mathrm{y}}^{2\omega}. \tag{29}$$

By fitting the experimental data on polarizations of the fundamental and second harmonic pulses we found the following initial parameters: $\varepsilon_{\omega} = 0.572$, $\varepsilon_{2\omega} = 0.301$, $\theta_{\omega} = 135^{\circ}$, $\theta_{2\omega} = 86^{\circ}$ (see Fig. 3a in the main text). The initial energies of the ω and 2ω pulses in our simulations are taken from the experiments and are equal to 8 and 0.8 mJ, respectively.

Together with the UPPE (see Supplementary Equation (19)) we solve the rate equation for the concentration of free electrons[5]:

$$\frac{\partial \rho}{\partial t} = R_1(\rho_{\mathrm{nt}} - \rho) + R_2 \rho, \tag{30}$$

where $\rho_{\mathrm{nt}} = 2.5 \times 10^{25}$ 1/m^3 is the concentration of neutral molecules, while R_1 and R_2 are the optical field and avalanche ionization rates. In the calculation of ρ, we assume that air consists of 79.1% of N_2 and 20.9% of O_2 molecules, with ionization potentials equal to 15.576 and 12.063 eV, respectively. The corresponding values of the multiphoton ionization orders K in Supplementary Equation (24) for 3.9 μm wavelength are 49 for N_2 and 38 for O_2. In our simulations we solve a separate rate equation for N_2 and O_2 molecules. For the calculation of $\partial\rho/\partial t$ in Supplementary Equation (24) we use only the first term on the right-hand side of Supplementary Equation (30). In turn, $\hat{J}_{\mathrm{A}i}$ is calculated as a sum of the corresponding expressions for N_2 and O_2.

To calculate the field ionization rate R_1 we use the Perelomov-Popov-Terentiev (PPT) model[12]. Since the PPT model has explicit analytical expressions only for the cases of linearly or circularly polarized light, in order to obtain R_1 for the elliptically polarized light, we use the following formula:

$$R_1 = (1 - \varepsilon_{\omega}) R_{1,\mathrm{linear}} + \varepsilon_{\omega} R_{1,\mathrm{circular}}, \tag{31}$$

where $R_{1,\mathrm{linear}}$ and $R_{1,\mathrm{circular}}$ are the field ionization rates for linearly and circularly polarized light given by the PPT model. Supplementary Figure 8 shows the field ionization rates R_1 of nitrogen N_2 and oxygen O_2 molecules for an electric field with linear, circular, and elliptic ($\varepsilon_{\omega} = 0.572$) polarizations.

The avalanche ionization rate R_2 for N_2 and O_2 molecules is given by

$$R_2 = \sigma(\omega_0) \frac{\left[E_{\mathrm{x}}^2 + E_{\mathrm{y}}^2\right]^{1/2}}{U_{\mathrm{i}}}, \tag{32}$$

with

$$\sigma(\omega_0) = \frac{q_e^2}{m_e} \frac{\nu_{\mathrm{c}}}{\nu_{\mathrm{c}}^2 + \omega_0^2}, \tag{33}$$

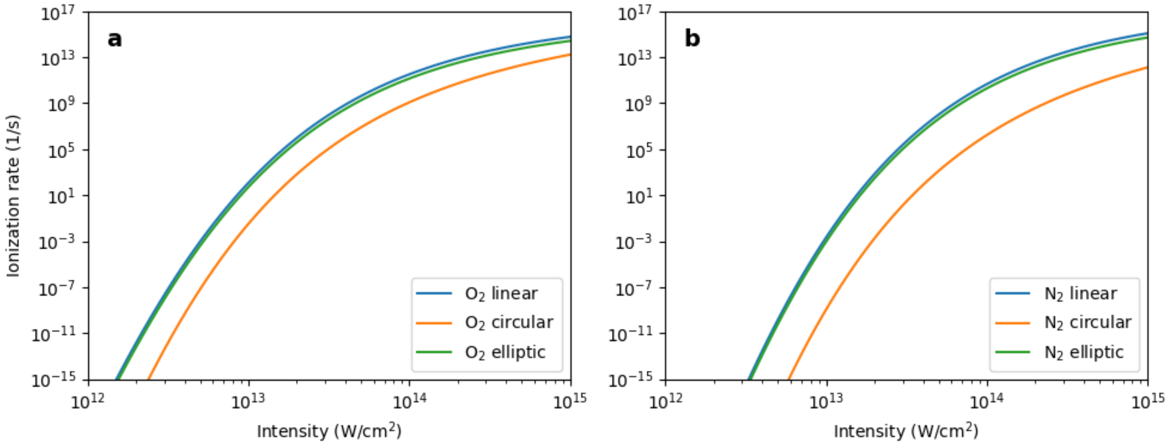

Supplementary Figure 8. Ionization rates of oxygen O_2 and nitrogen N_2 for different polarizations of the fundamental pulse. **a** Field ionization rates of O_2 and **b** N_2 for linearly, circularly, and elliptically ($\varepsilon_\omega = 0.572$) polarized fundamental pulses.

being the inverse Bremsstrahlung cross-section at the fundamental pulse central frequency ω_0, and U_i is the ionization potential.

We solved Supplementary Equation (19) on an axially symmetric grid with the following parameters: the grid size and number of points in the spatial domain are 12 mm and 1200, respectively (spatial resolution is 10 μm); the grid size and number of points in the time domain are 10 ps and 32768, respectively (temporal and spectral resolutions are 0.3 fs and 0.1 THz). In the spatial domain we use periodic boundary conditions in order to avoid numerical losses of fast diffracting THz radiation. On the both ends of the temporal grid we use absorbing layers of 250 fs width (the radiation inside these layers is multiplied by a smoothly decreasing function).

Supplementary References

1. Chang, L. *et al.* Waveguide-coupled micro-ball lens array suitable for mass fabrication. *Opt. Express* **23**, 22414 (2015).
2. Koulouklidis, A. D., Fedorov, V. Y. & Tzortzakis, S. Spectral bandwidth scaling laws and reconstruction of THz wave packets generated from two-color laser plasma filaments. *Phys. Rev. A* **93**, 033844 (2016).
3. Kolesik, M., Moloney, J. V. & Mlejnek, M. Unidirectional Optical Pulse Propagation Equation. *Phys. Rev. Lett.* **89**, 283902 (2002).
4. Kolesik, M. & Moloney, J. V. Nonlinear optical pulse propagation simulation: From Maxwell's to unidirectional equations. *Phys. Rev. E* **70**, 036604 (2004).
5. Couairon, A. *et al.* Practitioner's guide to laser pulse propagation models and simulation. *The Eur. Phys. J. Special Top.* **199**, 5–76 (2011).
6. Sprangle, P., Peñano, J., Hafizi, B. & Kapetanakos, C. Ultrashort laser pulses and electromagnetic pulse generation in air and on dielectric surfaces. *Phys. Rev. E* **69**, 066415 (2004).
7. Zahedpour, S., Wahlstrand, J. K. & Milchberg, H. M. Measurement of the nonlinear refractive index of air constituents at mid-infrared wavelengths. *Opt. letters* **40**, 5794–5797 (2015).
8. Mlejnek, M., Wright, E. & Moloney, J. Dynamic spatial replenishment of femtosecond pulses propagating in air. *Opt. letters* **23**, 382–384 (1998).
9. Panov, N. A. *et al.* Supercontinuum of a 3.9-μm filament in air: Formation of a two-octave plateau and nonlinearly enhanced linear absorption. *Phys. Rev. A* **94**, 041801 (2016).
10. Peck, E. R. & Reeder, K. Dispersion of Air. *J. Opt. Soc. Am.* **62**, 958–962 (1972).
11. HITRAN on the Web. http://hitran.iao.ru/. Accessed: 2017-07-05.
12. Perelomov, A. M., Popov, V. S. & Terent'ev, M. V. Ionization of atoms in an alternating electric field: II. *Sov. Phys. JETP* **24**, 207–217 (1967).
